# Supplementary material for: TGF-β activation by bone marrow-derived thrombospondin-1 causes Schistosoma- and hypoxia-induced pulmonary hypertension
Source: Nat Commun. 2017 May 30;8:15494. doi: 10.1038/ncomms15494 (PMC5459967; doi:10.1038/ncomms15494)
Supplement: Supplementary Information — Supplementary Figures and Supplementary Tables. [file ncomms15494-s1.pdf]

Supplementary Table 1: Left ventricular systolic pressure (LVSP), right and left ventricular diastolic pressures (RVDP/LVDP), heart rate (HR), and body weight (Bd. wt.) of IP/IV exposed and unexposed wildtype mice treated with either SLLK or LSKL peptide (mean ± SD; n=6, 5, 6 and 11 mice / group, respectively; ANOVA *P*=NS, non significant; IP/IV, Intraperitoneal/intravenous *S. mansoni* eggs.).

**Wildtype on Peptide**	**Experimental conditions**	**LVSP (mmHg)**	**RVDP (mmHg)**	**LVDP (mmHg)**	**HR (bpm)**	**Bd. wt. (g)**
SLLK	Unexposed	51.7±4.3	1.2±0.6	2.6±0.6	172.2±33.7	19.0±1.8
SLLK	IP/IV eggs	55.7±3.9	1.5±0.4	3.2±0.5	177.0±12.5	20.3±0.9
LSKL	Unexposed	56.4±6.9	1.3±0.5	2.6±0.8	170.2±21.7	19.9±0.9
LSKL	IP/IV eggs	57.1±4.2	1.8±0.6	3.2±0.5	160.1±26.2	20.6±1.2
ANOVA		NS	NS	NS	NS	NS

Supplementary Table 2: Left ventricular systolic pressure (LVSP), right and left ventricular diastolic pressures (RVDP/LVDP), heart rate (HR), and body weight (Bd. wt.) of IP/IV exposed and unexposed wildtype (WT) mice recipient of either wildtype or *Thbssp1* bone marrow (BM) (mean ± SD; n=10, 7 and 7 mice / group, respectively; ANOVA *P*=NS, non significant; IP/IV, Intraperitoneal/intravenous *S. mansoni* eggs.).

**BM Recipient**	**BM Donor**	**Experimental conditions**	**LVSP (mmHg)**	**RVDP (mmHg)**	**LVDP (mmHg)**	**HR (bpm)**	**Bd. wt. (g)**
WT	*Thbs1*$^{-/-}$	Unexposed	62.6±15.2	1.5±0.4	2.1±0.9	152±19.8	20.1±1.4
WT	*Thbs1*$^{-/-}$	IP/IV eggs	63.1±27.9	1.6±0.6	2.1±0.7	162.3±26.0	20.1±1.4
WT	WT	IP/IV eggs	73.6±10.5	1.2±0.7	1.5±0.7	188.7±75.3	19.0±1.8
ANOVA			NS	NS	NS	NS	NS

Supplementary Table 3: Left ventricular systolic pressure (LVSP), right and left ventricular diastolic pressures (RVDP/LVDP), heart rate (HR), and body weight (Bd. wt.) of chronic hypoxia (Ch. Hx) exposed and normoxic wildtpe mice treated with either SLLK or LSKL peptide (mean ± SD; n=6 mice / group; t test, *P*=NS, non significant).

**Wildtype on Peptide**	**Experimental conditions**	**LVSP (mmHg)**	**RVDP (mmHg)**	**LVDP (mmHg)**	**HR (bpm)**	**Bd. wt. (g)**
SLLK	Ch. Hx	74.9±5.4	1.7±0.8	2.3±0.9	272.2±62.7	17.6±0.9
LSKL	Ch. Hx	76.5±6.6	2.4±0.6	2.7±1.1	264.0±79.3	17.2±1.8
t-test		NS	NS	NS	NS	NS

Supplementary Table 4: Demographic and clinical characteristics of human scleroderma subjects, before and after diagnosis with pulmonary arterial hypertension. NHW, Non-Hispanic White; mPAP, mean Pulmonary Arterial Pressure; PVR, Pulmonary Vascular Resistance; TDCO, Thermodilution Cardiac Output; TDCI, Thermodilution Cardiac Index; RAP, Righ Arterial Pressure; FVC, Forced Vital Capacity; FEV_1, Forced Expiratory Volume in One Second; DLCO, Diffusing Capacity of the Lungs for Carbon Monoxide; 6MWT, 6 Minute Walk Test; NYHA, New York Heart Association; ND: not done (mean ± SD; number of samples with data available presented).

Parameters	SSc Pre-PAH	SSc Post-PAH
N	7	
Age	53.4±8.8	58.3±8.8
Gender	71% F	
Race/Ethnicity	71% NHW	
mPAP (mmHg)	20.0, 1	34.6±11.2, 7
PVR (Wood Units)	3.1, 1	5.4±3.3, 7
TDCO (L min^{-1})	4.6, 1	4.6±0.6, 7
TDCI (L min^{-1} m^{-2})	2.5, 1	2.7±0.2, 7
RAP (mmHg)	1.0, 1	6.0±3.9, 7
RVSP on Echo (mmHg)	31.8±8.0, 5	69.4±19.7, 7
FVC (% Predicted)	85.6±7.7, 5	77.2±13.3, 6
FEV1 (% Predicted)	81.0±8.0, 5	74.2±9.8, 6
DLCO (% Predicted)	61.8±22.9, 4	33.8±9.2, 6
6MWT	180, 1	268±87, 4
NYHA Functional Class	ND	2.3±0.5, 4

Supplementary Table 5: Reagents for quantifying mouse protein by ELISA.

Protein	Kit
TSP-1	Cloud-Clone Corp. SEA611Mu
IL-4	R&D Systems M4000B
IL-13	R&D Systems M1300CB
CCR2	LSBio LS-F19718
CCL2	R&D Systems MJE00
CCL7	MYBioSource MBS764604
CCL12	R&D Systems MCC120

Supplementary Table 6: Reagents for immunostaining mouse tissue. All rinses between steps in TBST.

Immunostain	Antigen Retrieval	Block	Primary Antibody	Secondary Antibody	Tertiary Reagent
αSM-actin	Borg Buffer 20 min in steamer (Biocare #BD1000G1)	Avidin 10 min, Biotin 10 min, Mouse on Mouse (MOM) kit blocking solution (Vector BMK-2202) 1hr at RT	1:200 (0.355 μg/ml) 30min at RT (Dako #M0851)	MOM Biotinylated anti-Mouse Reagent (Vector BMK-2202) 10min at RT, then 1:500 SA-Fluorescein (Invitrogen S869)	Vectashield with DAPI (Vector H-1500)
TSP-1			1:50 (2 μg/ml) 1hr at RT (abcam ab1823)		
Mac3		10% Rabbit Serum in 1:1 5% BSA: Superblock (ScyTek AAA5000) 1hr at RT	1:50 (0.625 μg/ml) 1hr at RT (BD Pharmingen #550292)	1:200 AF594 Rabbit anti-Rat (Invitrogen #A21211) 1hr at RT	
Thrombo-modulin (CD141)		10% Horse Serum in 1:1 5% BSA: Superblock 1hr at RT	1:200 (1 μg/ml) 1hr at RT (R&D Systems #AF3894)	1:200 AF594 Donkey anti-Goat (Invitrogen #A11058) 1hr at RT	

Supplementary Table 7: Antibodies used in the flow cytometry experiment.

Antibody Specificity	Fluochrome	Isotype	Clone	Final Concenration	Manufacturer
Anti-mouse TSP-1	PE	IgG2a Kappa	TX17.10	0.2µg/10^6 cells	Novus
Anti-mouse CD64	BV711	Mouse IgG1, κ	X54-5/7.1	0.1µg/10^6 cells	Biolegend
Mouse MerTK Biotinylated Antibody	Biotin-PE-Cy7	IgG	-	1µg/10^6 cells	R&D Systems
Anti-mouse/Rat CD11b	AF700	IgG2b, kappa	M1/70	0.1µg/10^6 cells	eBioscience®
Anti-mouse CD11c	PerCp Cy5.5	IgG	N418	0.1µg/10^6 cells	eBioscience®
Anti-mouse Ly6C	APC	IgG2c, kappa	HK1.4	0.1µg/10^6 cells	eBioscience®

Supplementary Table 8: Reagents for immunostaining human tissue. All rinses between steps in PBS.

Immuno-stain	Antigen Retrieval	Block	Primary Antibody	Secondary Antibody	Tertiary Reagent
CD68	Borg Buffer 20 min in steamer (Biocare #BD1000G1)	10% Horse Serum in PBS 1hr at RT	1:100 (0.2 µg/ml) in PBS O/N at 4degC (ThermoFisher #PA5-32331)	1:200 AF594 Donkey anti-Rabbit (Invitrogen #A21207) 1hr at RT	Vectashield with DAPI (Vector H-1500)
vWF			1:8,000 (0.388 µg/ml) in PBS O/N at 4degC (Dako #A0082)		
TSP-1			1:50 (2 µg/ml) in PBS O/N at 4degC (abcam ab1823)	1:200 AF488 Donkey anti-Mouse (Invitrogen #A21202) 1hr at RT	

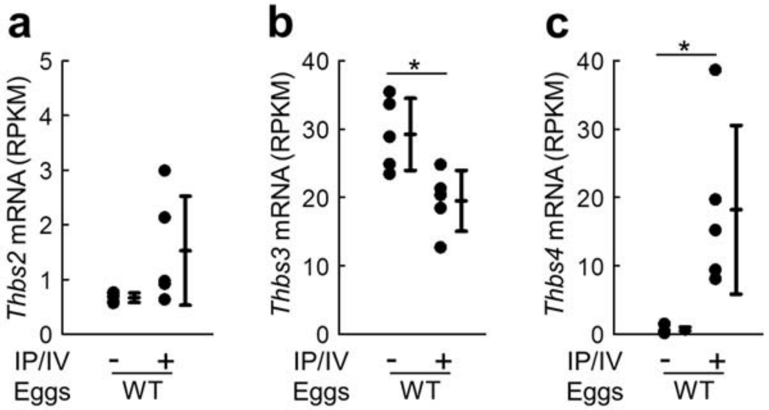

Supplementary Figure 1. mRNA quantity of other thrombospondin isoforms. Whole lung concentrations of *Thbs2*, *Thbs3* and *Thbs4* mRNA by RNA-seq (n=5 mice / group; mean ± SD plotted; RPKM: reads per kilobase per million mapped reads; t-test; **P*<0.05) in mice unexposed or with *Schistosoma*-induced PH. IP/IV=intraperitoneal and intravenous *S. mansoni* eggs.

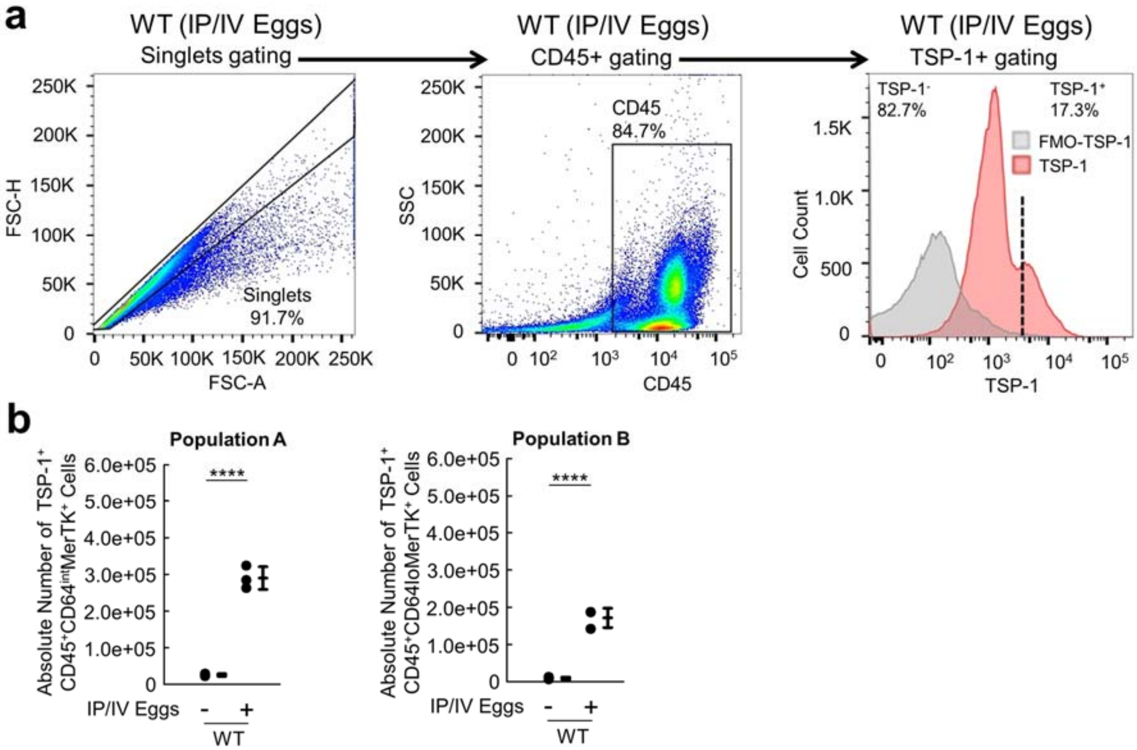

Supplementary Figure 2. Schematic representation of flow cytometry analysis of TSP-1$^+$ cells in *Schistosoma*-exposed mice. (**a**) Gating strategy for flow cytometry: singlets were gated for CD45$^+$ cells, followed by identifying the TSP-1+ gate (threshold determined by FMO for each sample; representative image of 3 / group). (**b**) Absolute numbers of the A and B populations from Figure 1c (n=3 / group; mean ± SD plotted; t-test, ****P<0.001). FSC, forward scatter; SSC, side scatter; IP/IV=intraperitoneal and intravenous *S. mansoni* eggs.

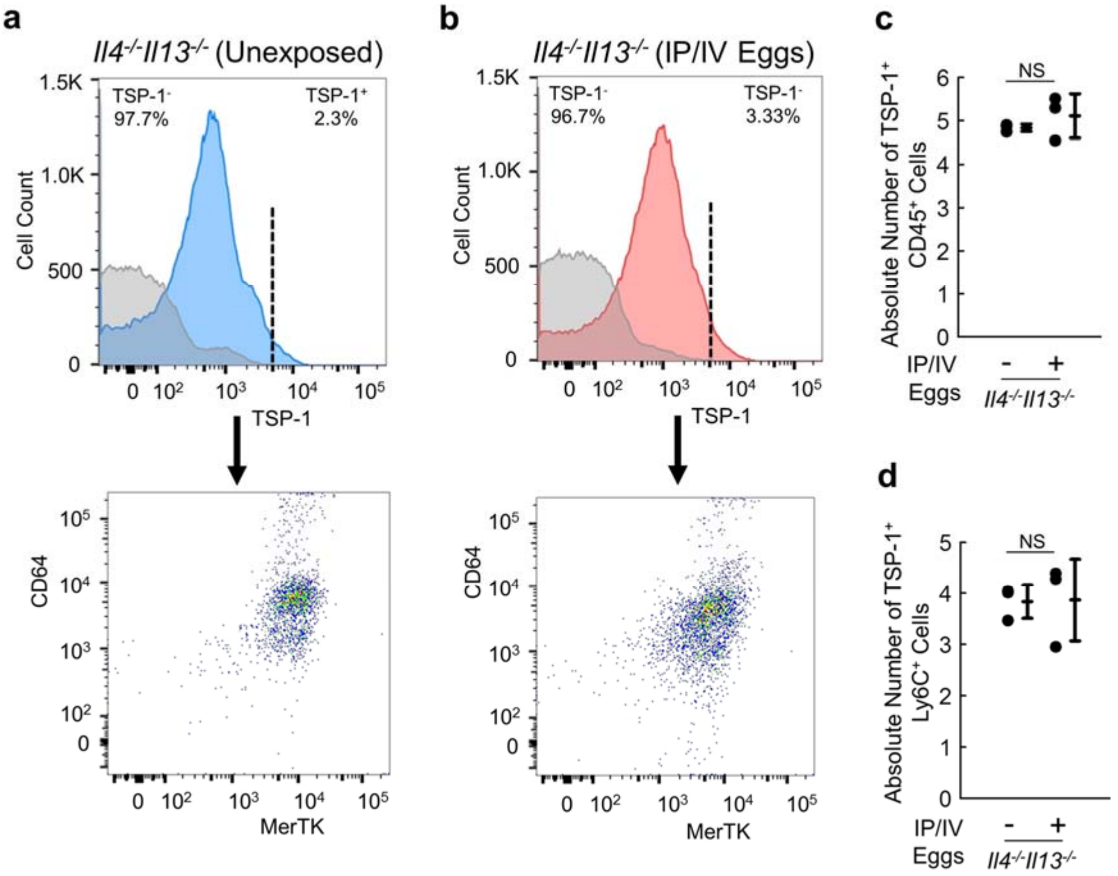

Supplementary Figure 3. Flow cytometry analysis in unexposed and *Schistosoma*-exposed *Il4$^{-/-}$Il13$^{-/-}$* mice. Representative histograms from cell-dispersed whole lung tissue, which stain positive for intracellular TSP-1 by flow cytometry from (**a**) unexposed or (**b**) *Schistosoma*-exposed *Il4$^{-/-}$Il13$^{-/-}$* mice. Log transformed (**c**) absolute number of CD45+TSP-1+ populations and (**d**) absolute number of CD45+Ly6C+ populations (n=3 / group; mean ± SD plotted, t test, NS=non-significant; IP/IV = intraperitoneal and intravenous *S. mansoni* eggs).

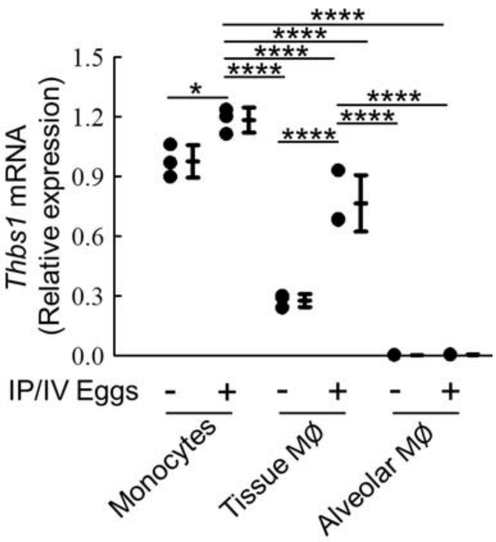

Supplementary Figure 4. *Thbs1* mRNA in sorted Ly6C$^+$ monocytes, tissue macrophages (MØ), and alveolar macrophages (MØ). *Thbs1* mRNA transcript in sorted cells from unexposed or *Schistosoma*-exposed digested mouse lungs. The sorted cell populations for monocytes are as shown in Figure 1c, tissue MØ are CD11b$^+$CD11c$^-$, and alveolar MØ are CD11c$^+$SiglecF$^+$, respectively. (Pooled mRNA from n=3 mice / group; mean of 3 technical replicates per group shown; $2^{-\Delta Ct}$ relative to *HPRT* housekeeping gene; mean ± SD plotted; t-test; *P<0.05, ****P<0.001). IP/IV=intraperitoneal and intravenous *S. mansoni* eggs.

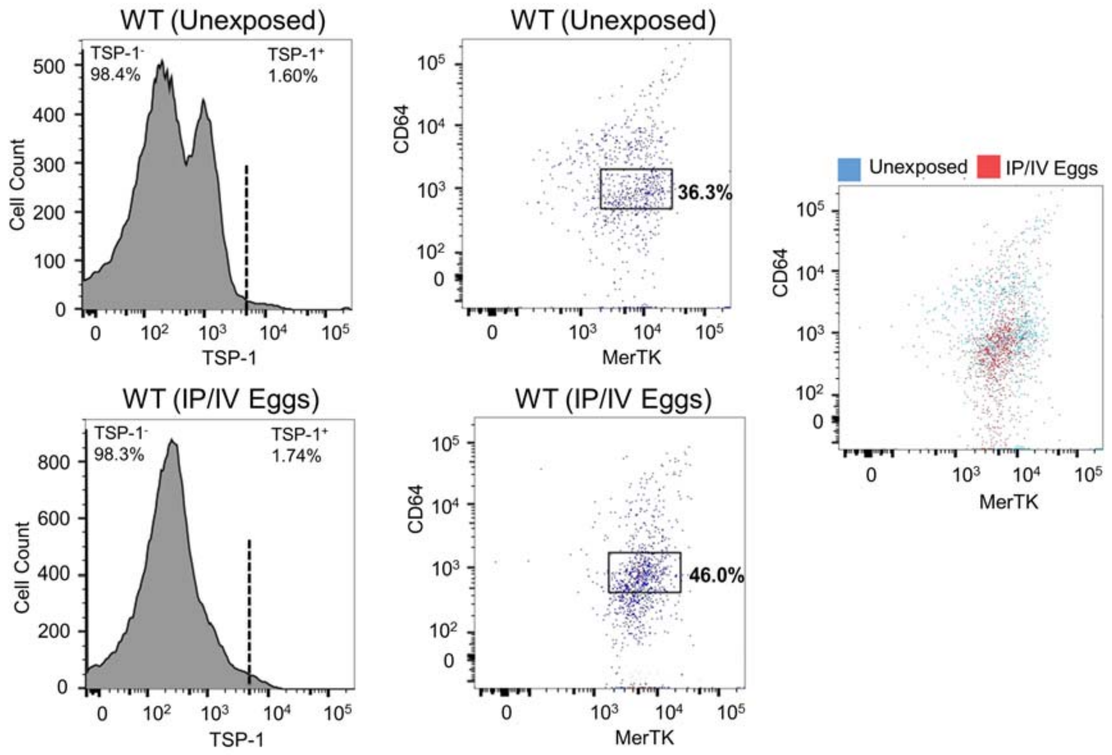

Supplementary Figure 5. Flow cytometry analysis of extracellular TSP-1 staining in *Schistosoma* exposed mice. Flow cytometry of cells from cell-dispersed whole lung tissue with extracellular-only staining of TSP-1, followed by the same gating strategy as in Figures 1B and C, in *Schistosoma* exposed and unexposed wildtype mice. Flow studies representative of at least 3 experiments / group; IP/IV=intraperitoneal and intravenous *S. mansoni* eggs.

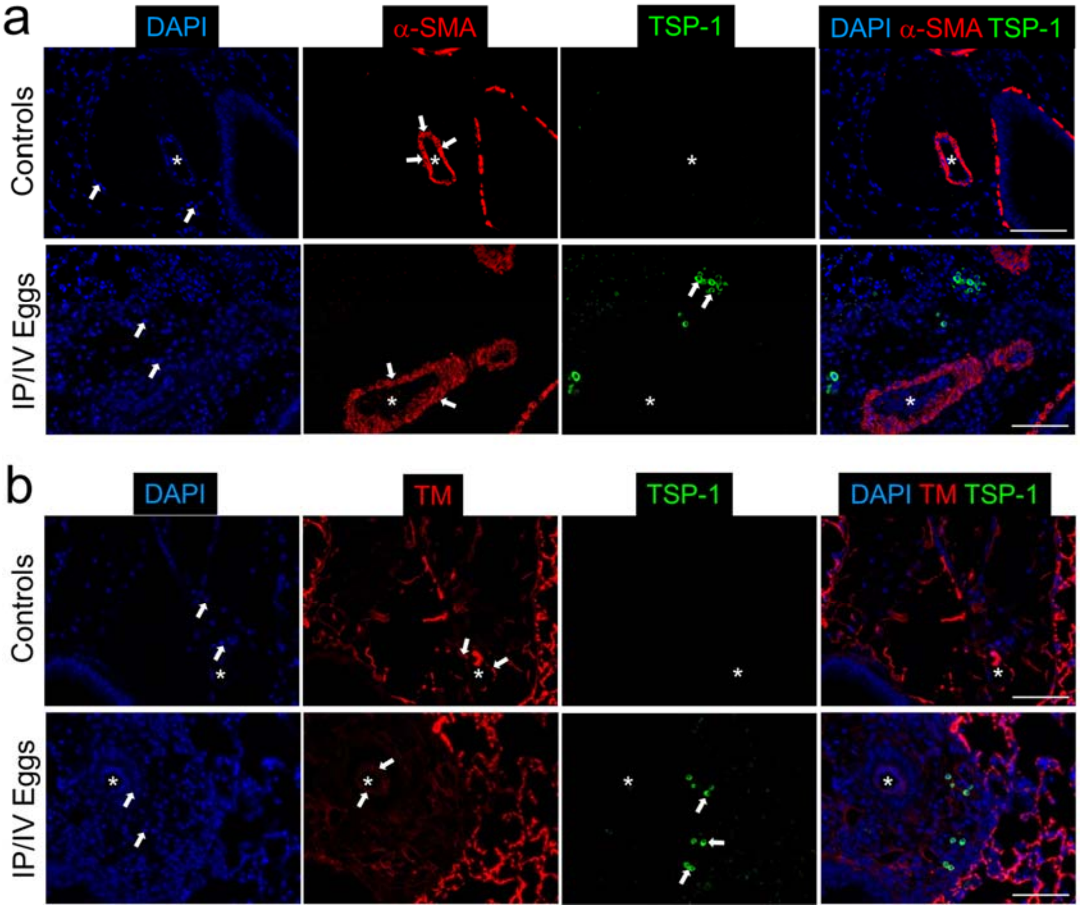

Supplementary Figure 6. No evidence of TSP-1 colocalization with the pulmonary vascular media or intima in *Schistosoma* egg unexposed and exposed wildtype mice. (**a**) Co-staining for α-SMA (smooth muscle cell marker) and TSP-1 in *Schistosoma* eggs unexposed and exposed wildtype mice. (**b**) Co-staining for TM (endothelial cell marker) and TSP-1 in *Schistosoma* eggs unexposed and exposed wildtype mice (arrows show areas of either DAPI, SMA, TM or TSP-1^{+} staining). DAPI, 4',6-diamidino-2-phenylindole, SMA, smooth muscle actin; TM, thrombomodulin (*: vessel lumen; all scale bars 100μm.). IP/IV= intraperitoneal and intravenous *S. mansoni* eggs.

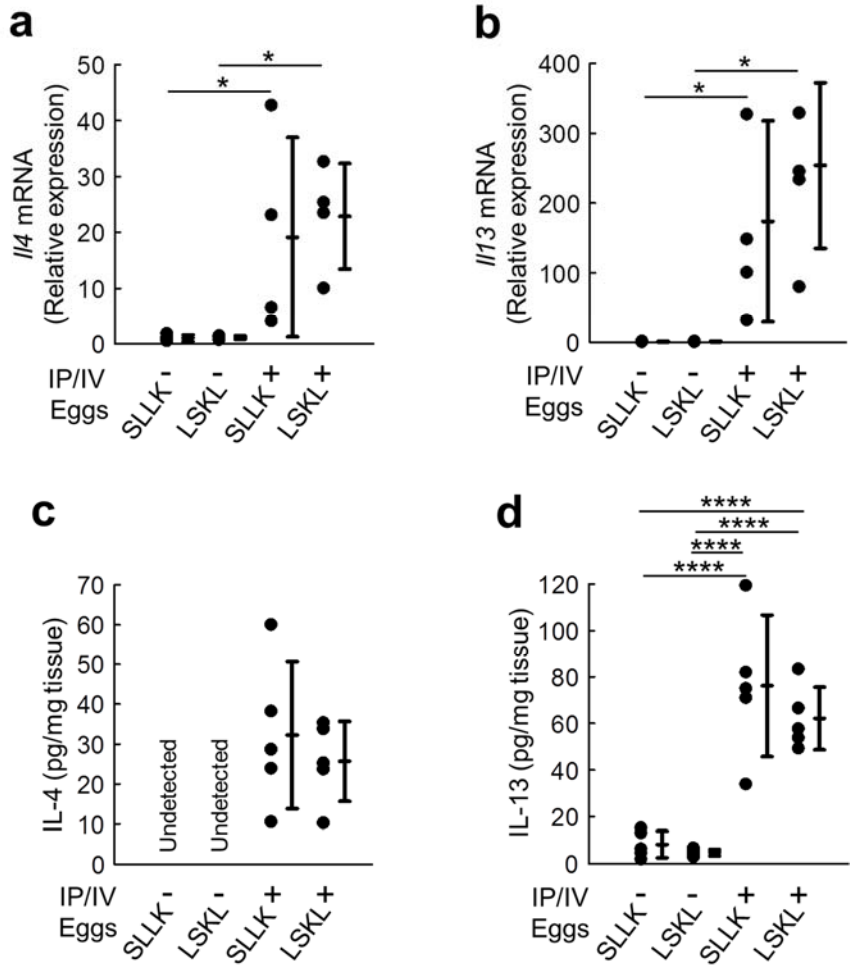

Supplementary Figure 7. IL-4 and IL-13 levels are unchanged in TSP-1 blockade. mRNA level of (**a**) *Il4* (n=4 / group) and (**b**) *Il13* (n=3, 3, 4, 4 / group, respectively); and protein levels of (**c**) IL-4 (n=5 / group) and (**d**) IL-13 (n=5 / group) in whole lung lysates of SLLK or LSKL treated mice, either unexposed or exposed to IP/IV eggs (mean ± SD plotted, mRNA quantified by RT-PCR; $2^{-\Delta Ct}$ relative to the geometric mean of β-actin and *Gapdh* housekeeping genes; normalized to mean of SLLK unexposed group = 1; ANOVA *P*<0.05, each, with *post-hoc* Tukey tests shown). IP/IV=intraperitoneal and intravenous *S. mansoni* eggs.

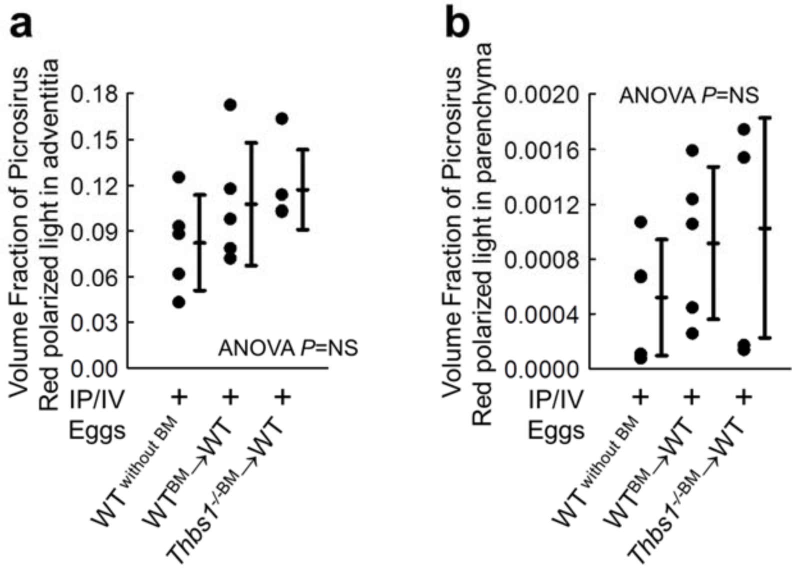

Supplementary Figure 8. No evidence of perivascular and parenchymal fibrosis in irradiated mice followed by bone marrow (BM) transplant. Quantitaive analysis using picrosirius red staining showed no difference in the volume fraction of (**a**) vascular adventitia and (**b**) parenchyma, which contains polarized material (i.e., Type 1 collagen) among wildtype mice without BM transplant, or receiving BM from WT or *Thbs1*$^{-/-}$ mice. (n=5/group; mean ± SD plotted; ANOVA *P* value: NS, non-significant). IP/IV=intraperitoneal and intravenous *S. mansoni* eggs.

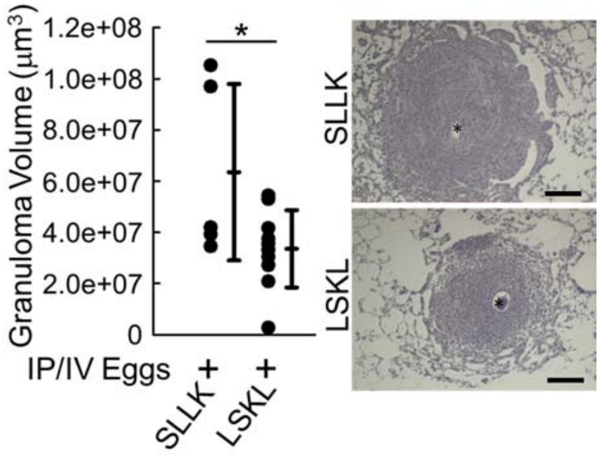

Supplementary Figure 9. Granuloma volumes following TSP-1 blockade. LKSL or SLLK treatment of wildtype, following IP/IV egg-exposure (n=5 and 10 mice / group, respectively; mean ± SD plotted; t-test; **P*<0.05, *: vessel lumen; scale bars: 100μm). IP/IV=intraperitoneal and intravenous *S. mansoni* eggs.

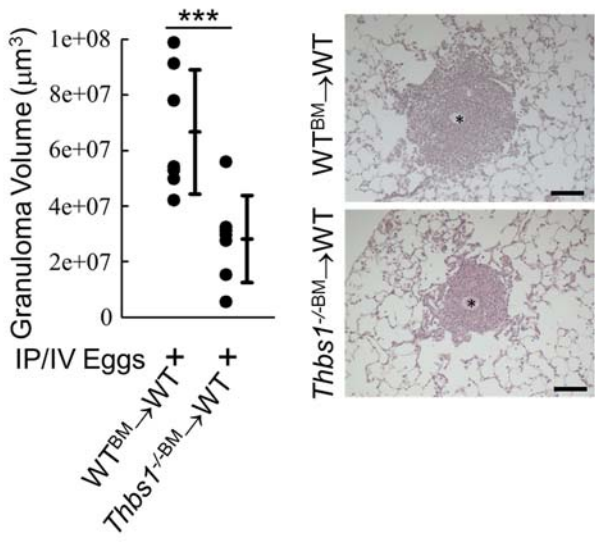

Supplementary Figure 10. Granuloma volumes in *Thbs1*$^{-/-}$ bone marrow-deficient mice. Wildtype (WT) recipients of wildtype or *Thbs1*$^{-/-}$ bone marrow, following IP/IV egg-exposure (n=7 mice / group; mean ± SD plotted; t test, **P*<0.005, *: vessel lumen; scale bars: 100μm). IP/IV=intraperitoneal and intravenous *S. mansoni* eggs.

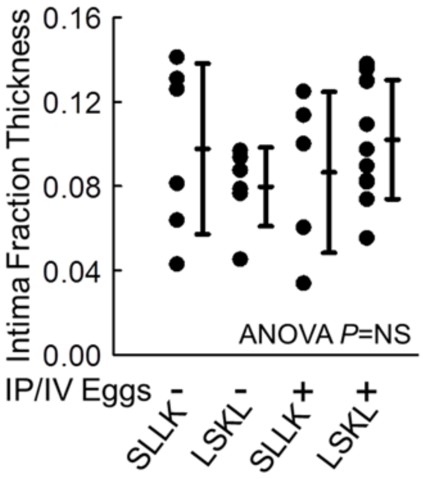

Supplementary Figure 11. No change in the pulmonary vascular intima thickness in *Schistosoma*-exposed wildtype mice treated with either scrambled (SLLK) or LSKL peptide. (n=6, 6, 5 and 11 mice / group, respectively; mean ± SD plotted; ANOVA *P*=NS). NS=non-significant, IP/IV=intraperitoneal and intravenous *S. mansoni* eggs.

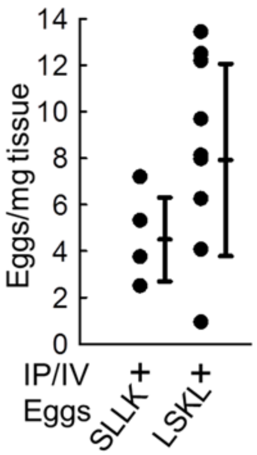

Supplementary Figure 12. Egg burden 1 week after IV egg augmentation following TSP-1 blockade. LSKL or SLLK treatment of wildtype mice, following IP/IV egg-exposure (N=5 and 10 mice / group; mean ± SD plotted; t test, *P*=NS). IP/IV=intraperitoneal and intravenous *S. mansoni* eggs.

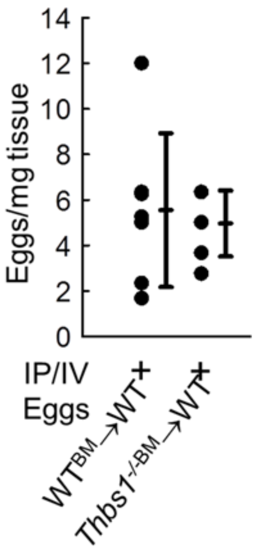

Supplementary Figure 13. Egg burden 1 week after IV egg augmentation in *Thbs1$^{-/-}$* bone marrow-deficient mice. Wildtype (WT) recipients of wildtype or *Thbs1$^{-/-}$* bone marrow, following IP/IV egg-exposure (N=7 and 6 mice/group, respectively; mean ± SD plotted; t test, *P*=NS). IP/IV=intraperitoneal and intravenous *S. mansoni* eggs.

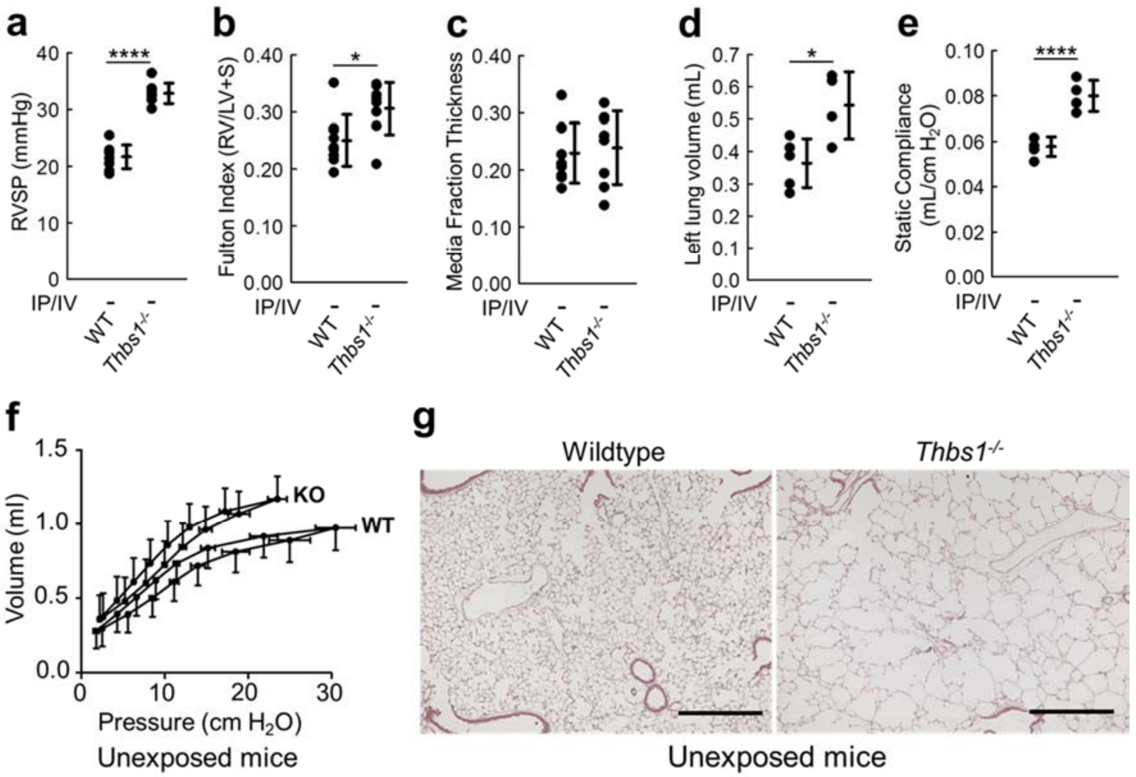

Supplementary Figure 14: *Thbs1$^{-/-}$* mice have at baseline higher right ventricular systolic pressure (RVSP) and emphysema. IP/IV eggs unexposed *Thbs1$^{-/-}$* mice showed higher baseline (**a**) RVSP (n=9 and 8 mice/group, respectively; mean ± SD plotted; t test, *****P*<0.001); (**b**) Fulton index (RV /(LV+S)) (n=9 and 8 mice/group, respectively; mean ± SD plotted; t test, **P*<0.05); with no diference in (**c**) media thickness (n=9 and 8 mice/group, respectively; mean ± SD plotted; t-test *P*=NS). *Thbs1$^{-/-}$* mice did have (**d**) larger left lung volumes (as measured by water displacement) (n= 5 and 5 mice/group, respectively; mean ± SD plotted; t test, **P*<0.05), (**e** and **f**) increased static compliance by pulmonary function testing (n=4 mice / group; mean ± SD plotted; t test, *****P*<0.001 in **e**; mean ± SEM for pressures and volumes displayed on graph in **f**), and (**g**) emphysema on lung histology as compared to wildtype (WT) mice (representative image of n=5 / group; scale bar: 500 µm). IP/IV=intraperitoneal and intravenous *S. mansoni* eggs.

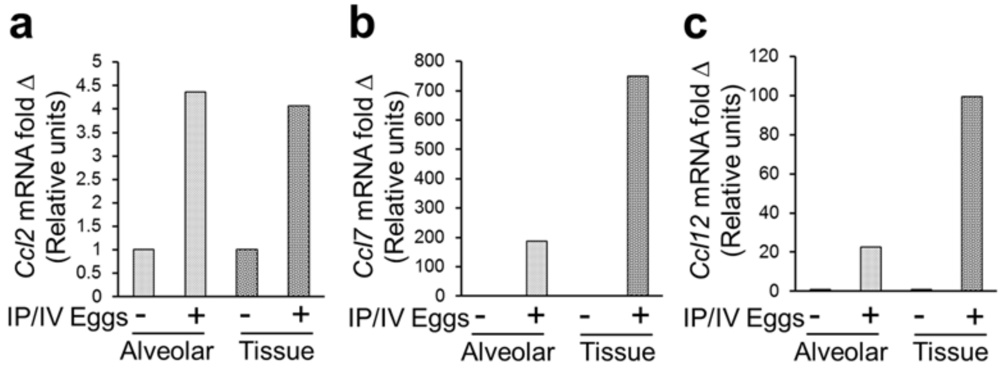

Supplementary Figure 15. Evidence of higher expression of the CCR2 ligands CCL2, CCL7 and CCL12 in sorted tissue macrophages from *Schistosoma*-exposed mice. RNA-seq analysis of sorted tissue macrophages (CD45^{+}F4/80^{+}CD11b^{+}SSC^{low}) versus alveolar macrophages (CD45^{+}F4/80^{+}CD11c^{+}SiglecF^{+}) from IP/IV egg-exposed or unexposed wildtype mice to determine transcript quantities of (**a**) *Ccl2*, (**b**) *Ccl7*, and (**c**) *Ccl12* (pooled mRNA from 3 mice / group; normalized to alveolar unexposed = 1). IP/IV=intraperitoneal and intravenous *S. mansoni* eggs.

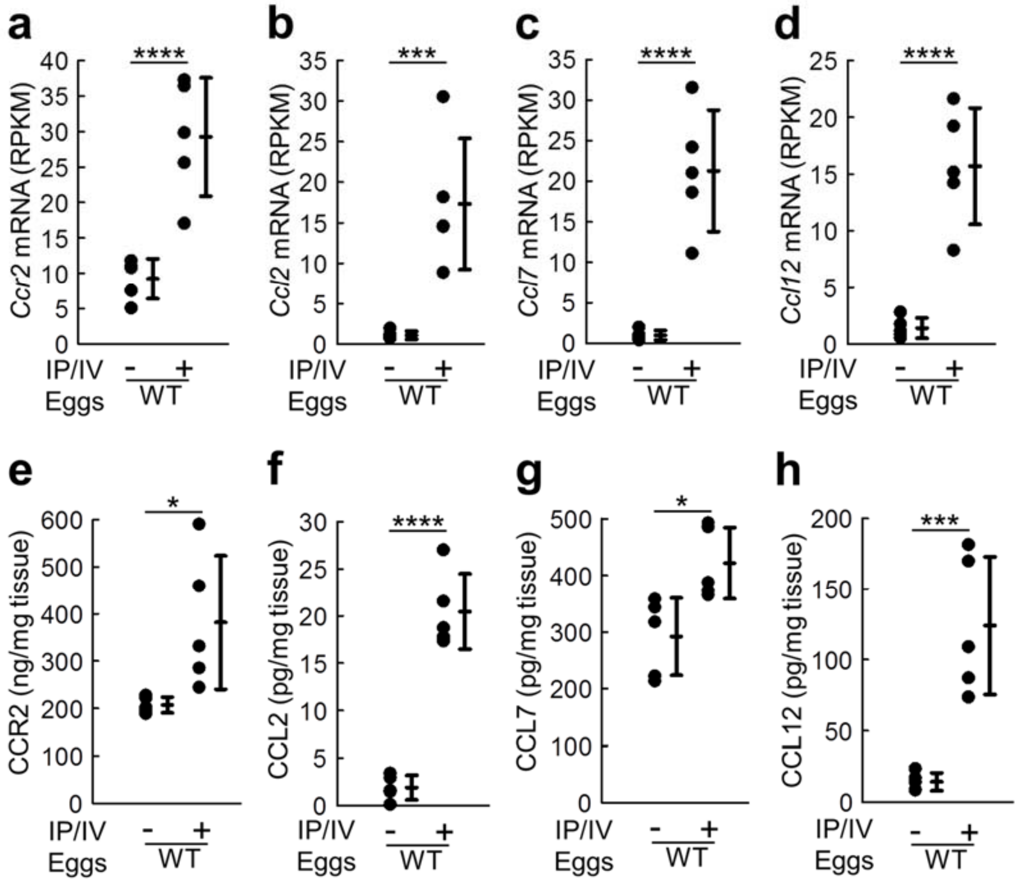

Supplementary Figure 16. Evidence of higher expression of CCR2 (receptor on Ly6C$^+$ monocytes for recruitment) and its ligands in whole lung lysates of *Schistosoma* exposed wildtype mice. RNA seq analysis for mRNA transcript quantity of (**a**) *Ccr2*, (**b**) *Ccl2*, (**c**) *Ccl7*, and (**d**) *Ccl12* and protein concentration of (**e**) CCR2, (**f**) CCL2, (**g**) CCL7, and (**h**) CCL12 in whole lung tissue of wildtype IP/IV egg-exposed and unexposed, (for each, n=5 samples/group; RPKM: reads per kilobase per million mapped reads; mean ± SD plotted; t-test; *P<0.05, ***P<0.005, ****P<0.001). IP/IV=intraperitoneal and intravenous *S. mansoni* eggs.

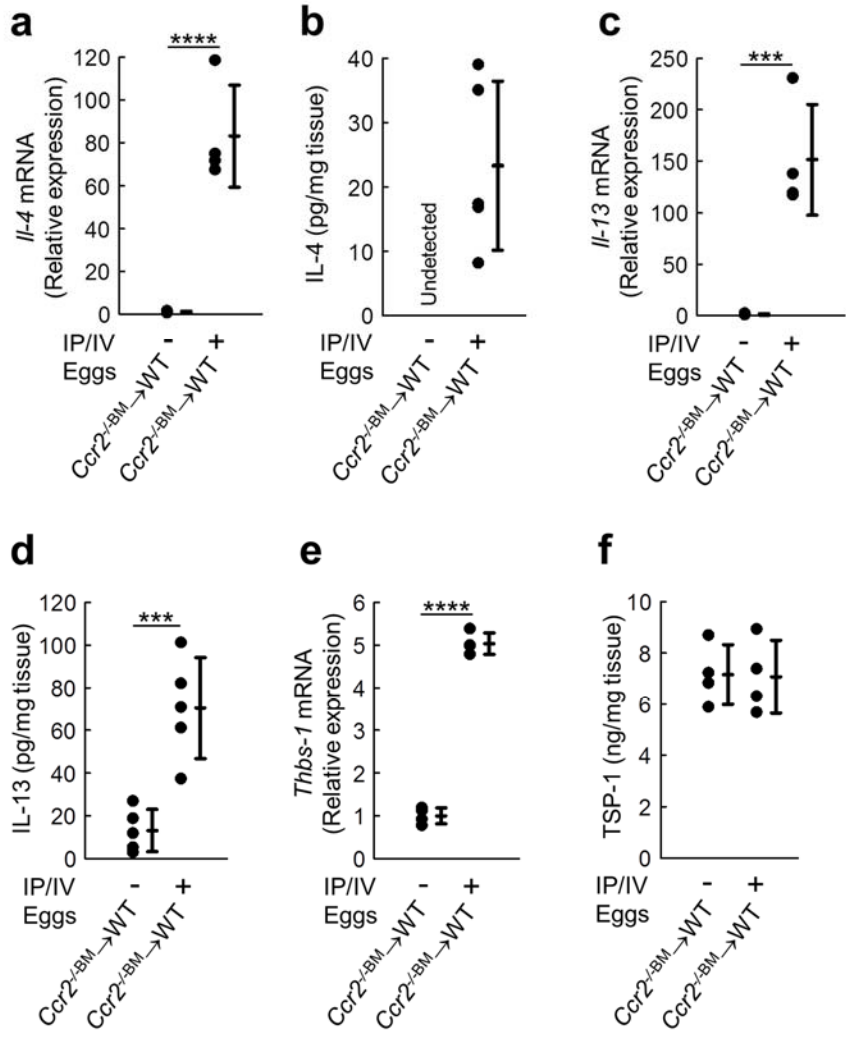

Supplementary Figure 17. IL-4, IL-13 and TSP-1 levels are higher in *Schistosoma* exposed wildtype recipient of *Ccr2*$^{-/-}$ bone marrow (BM) cells. mRNA and protein levels of (**a&b**) *Il4*/IL-4, (**c&d**) *Il13*/IL-13 and (**e&f**) *Thbs1*/TSP-1 in whole lung lysates of *Schistosoma* egg exposed and unexposed wildtype mice recipient of *Ccr2*$^{-/-}$ BM (n=4, 4; 5, 5; 4, 4; 5, 5; 4, 4; 4 and 4 /group, respectively; mean ± SD plotted; mRNA quantified by RT-PCR; $2^{-\Delta Ct}$ relative to the geometric mean of β-actin housekeeping gene; normalized to mean of wildtype recipient of *Ccr2*$^{-/-}$ BM unexposed group = 1; t-tests shown, ***P<0.005, ****P<0.001). IP/IV=intraperitoneal and intravenous *S. mansoni* eggs.

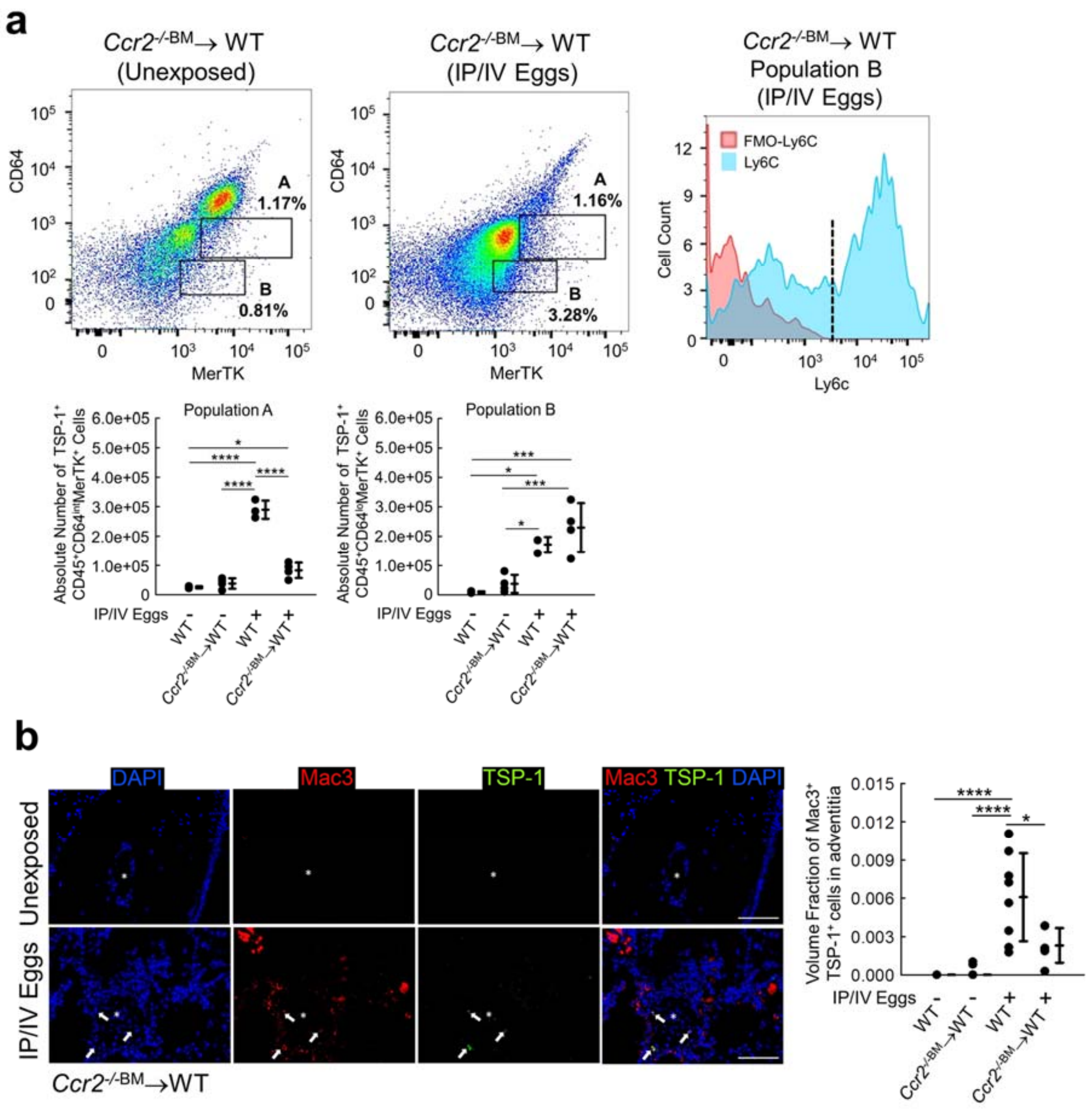

Supplementary Figure 18. Evidence of less recruitment of Ly6C+ cells in wildtype mice recipient of *Ccr2*$^{-/-}$ bone marrow (BM) cells and TSP-1^{+}Mac3^{+} co-localization. (**a**) Flow cytometry was performed on WT recipients of *Ccr2*$^{-/-}$ BM (as compared to the WT mouse data reproduced here from Figure 1c). Gating on the CD45^{+}TSP-1^{+} population (using the same gating strategy as in wildtype mice in Supplementary Fig. 2 above) in wildtype recipients of *Ccr2*$^{-/-}$ BM unexposed and *Schistosoma*-exposed indicates a increase in CD64^{lo}MerTK^{+}Ly6C^{+} cells (population "B"), consistent with intravascular monocytes, but suppression of the CD64^{int}MerTK^{+} cells (population "A") which are consistent with monocytes recruited into the parenchyma (n=3/group for WT and

n=4/group for WT recipients of *Ccr2*$^{-/-}$ BM). (**b**) Quantification of the volume fraction of TSP-1$^+$Mac3$^+$ cells in the adventitia of vessels by stereology; colocalization data for *Schistosoma* exposed and exposed wildtype mice without BM transplantation and wildtype recipient of *Ccr2*$^{-/-}$ BM. The wildtype data without BM transplantation is same as shown in Figure 1E) (asterisk: vessel lumen; arrows: representative positive double-stained cells; scale bars: 100 µm; n=5, 6, 8 and 6 mice / group, respectively; mean ± SD plotted; ANOVA *P*<0.001 with *post-hoc* Tukey tests shown; **P*<0.05; ****P*<0.005, *****P*<0.001). IP/IV=intraperitoneal and intravenous *S. mansoni* eggs.

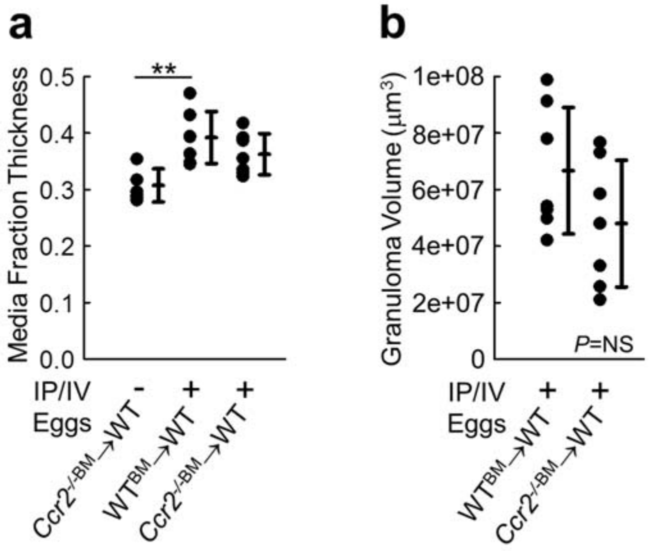

Supplementary Figure 19: Mildly depressed media thickness, and no change in intima thickness or granuloma volumes between *Schistosoma* exposed wildtype mice recipient of CCR2$^{-/-}$ and wildtype bone marrow (BM) cells. (**a**) Media fractional thickness (n=5, 7, 7 and mice / group, respectively) and (**b**) peri-egg granuloma volumes (n=7 mice / group, respectively), in unexposed or IP/IV eggs-exposed wildtype recipients of *Ccr2*$^{-/-}$ BM mice compared to wildtype recipients of wildtype BM (mean ± SD plotted; ANOVA *P*=0.006 with *post-hoc* Tukey tests shown, ***P*<0.01). IP/IV=intraperitoneal and intravenous *S. mansoni* eggs. The WT^{BM} → WT data is the same as in Figure 2b (media fractional thickness) and Supplementary Figure 10 (granuloma volumes).

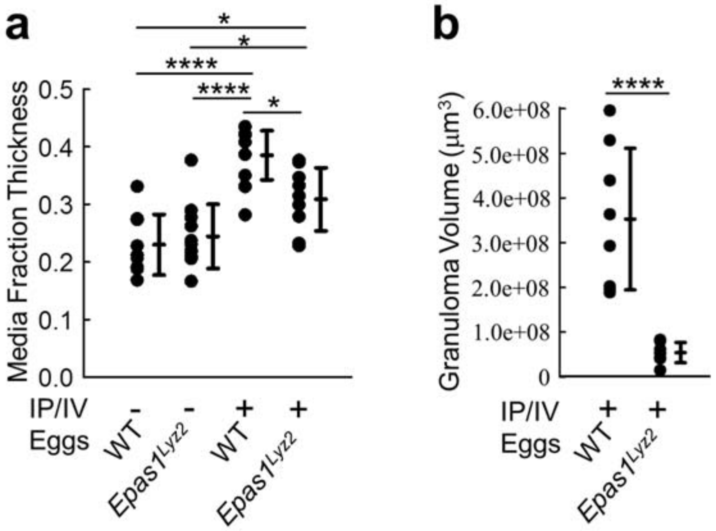

Supplementary Figure 20. Less vascular remodeling and smaller granulomas in *Epas1$^{fl/fl}$* x *Lyz2-Cre* mice following *Schistosoma* exposure. (a) Quantitative fractional thickness of the pulmonary vascular media of unexposed or *Schistosoma*-exposed wildtype (WT) and *Epas1$^{fl/fl}$* x *Lyz2-Cre* mice (representated as *Epas1^{Lyz2}*) (n=9, 11, 9 and 9 mice / group, respectively; mean ± SD plotted; ANOVA *P*<0.001, with *post hoc* Tukey tests shown). **(b)** Peri-egg granuloma volumes of *Schistosoma*-exposed wildtype (WT) and *Epas1$^{fl/fl}$* x *Lyz2-Cre* mice (n=8 mice/group; mean ± SD plotted; t test ; *P* values: *P<0.05; ****P<0.001). IP/IV=intraperitoneal and intravenous *S. mansoni* eggs.

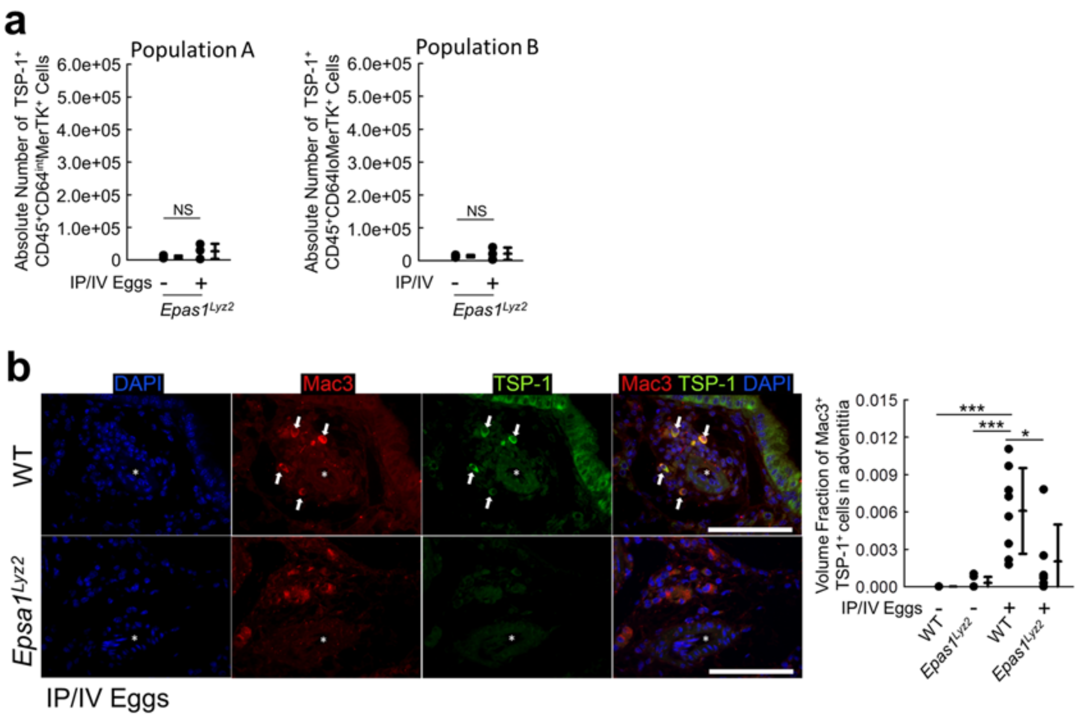

Supplementary Figure 21. Evidence of Hif2α regulation of TSP-1 in myeloid cells. (**a**) Absolute numbers of the A and B populations from Figure 3c (N=3/group; mean ± SD plotted; t-test) in *Schistosoma* exposed and unexposed *Epas1$^{fl/fl}$* x *Lyz2-Cre* mice. (**b**) Representative immunostaining for TSP-1 and Mac3 (macrophage marker), and quantification of the volume fraction of TSP-1^{+}Mac3^{+} cells in the adventitia of vessels by stereology; colocalization data for *Schistosoma* exposed and exposed wildtype mice and *Epas1$^{fl/fl}$* x *Lyz2-Cre* are same as shown in Figure 1E and Figure 3D, reproduced here for comparison between wildtype and the *Epas1$^{fl/fl}$* x *Lyz2-Cre* data (asterisk: vessel lumen; arrows: representative positive double-stained cells; scale bars: 100 μm; n=5, 6, 8 and 6 mice/ group, respectively; ANOVA *P*<0.001 with *post-hoc* Tukey tests shown). (mean ± SD plotted; *P* values: NS, non-significant; **P*<0.05; ****P*<0.005). IP/IV=intraperitoneal and intravenous *S. mansoni* eggs.

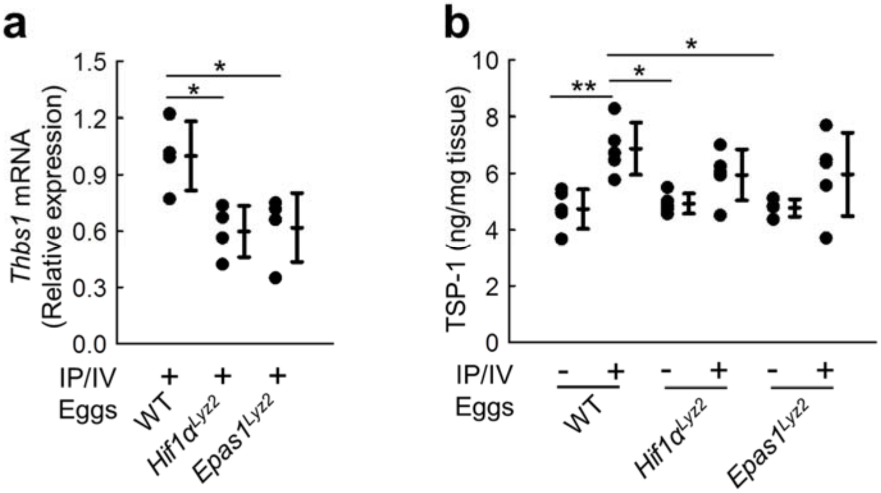

Supplementary Figure 22. Evidence of both Hif1α and Hif2α regulation of TSP-1 in myeloid cells. Whole lung (**a**) *Thbs1* mRNA by RT-PCR in *Schistosoma*-exposed (n-4/group) and (**b**) TSP-1 protein by ELISA in *Schistosoma*-exposed and unexposed wildtype, *Hif1a*$^{fl/fl}$ x *Lyz2-Cre* and *Epas1*$^{fl/fl}$ x *Lyz2-Cre* mice (n= 5, 5, 5, 5, 4 and 5 mice / group, respectively). ($2^{-\Delta Ct}$; relative to β-actin housekeeping gene; mean ± SD plotted; ANOVA P=0.014 with *post-hoc* Tukey tests shown, *P<0.05, **P<0.01). IP/IV=intraperitoneal and intravenous *S. mansoni* eggs.

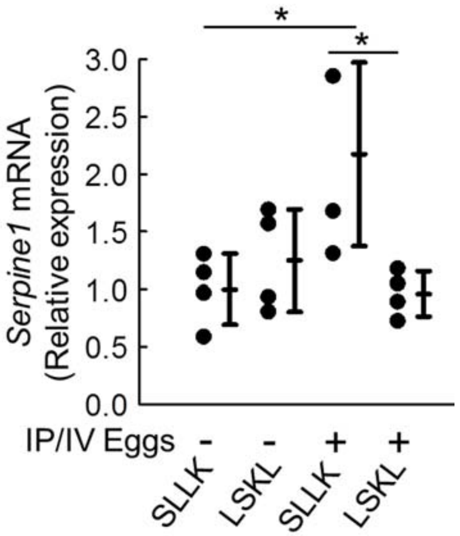

Supplementary Figure 23: LSKL treated mice showed significant reduction in TGF-β target gene *Serpine1*/*Pai1* as measured by RT-PCR (n=4/group; mRNA quantified by RT-PCR; $2^{-\Delta Ct}$ relative to the geometric mean of β-actin housekeeping gene; normalized to mean of wildtype SLLK treated unexposed group = 1; mean ± SD plotted; ANOVA *P*<0.015 with *post-hoc* Tukey tests shown, **P*<0.05). IP/IV=intraperitoneal and intravenous *S. mansoni* eggs.

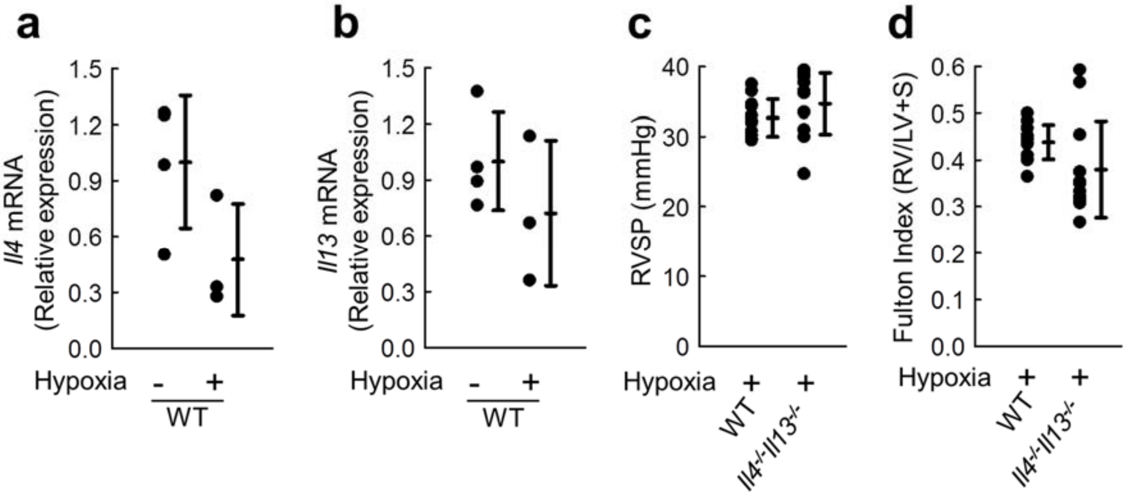

Supplementary Figure 24. No evidence for a role for Type 2 inflammation in chronic hypoxia-induced PH. (**a-b**) *Il4* and *Il13* mRNA quantity in normoxia or chronic hypoxia-exposed mice by RT-PCR (n=4 mice / group; $2^{-\Delta Ct}$; relative to β-actin housekeeping gene; mean ± SD plotted; t-test *P*=NS for both). (**c-d**) Right ventricular systolic pressure (RVSP) (n=13 and 12 mice / group, respectively), and Fulton index (n=13 and 12 mice/ group, respectively) in wildtype (WT) and *Il4*$^{-/-}$*Il13*$^{-/-}$ mice exposed to chronic hypoxia (t-test, *P*=NS, non significant for both; mean ± SD plotted).

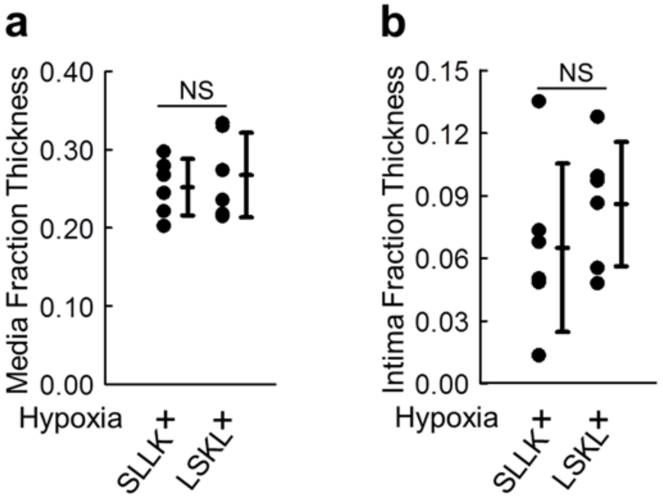

Supplementary figure 25. No change in pulmonary vascular media or intima remodeling in hypoxia exposed wildtype mice treated with either scrambled (SLLK) or LSKL peptide. (n=6 mice / group; t-test, *P*=NS, non significant for both; mean ± SD plotted).

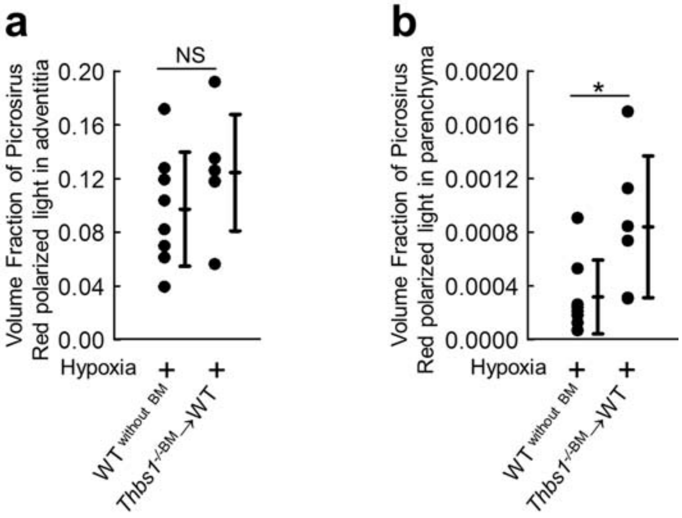

Supplementary Figure 26. No evidence of perivascular fibrosis, but mild increase in parenchymal fibrosis in irradiated mice for by bone marrow (BM) transplant followed by hypoxa exposure. Quantitaive analysis using Picrosirius red staining showed no difference in the volume fraction of (**a**) vascular adventitia with (**b**) a partial upregulation in parenchyma, positive for Picrosirus red polarization between hypoxia exposed wildtype mice or wildtype mice receiving *Thbs1*$^{-/-}$ bone marrow (BM; n=8, 6; 8 and 8 mice /group, resepectively; mean ± SD plotted; t-test, *P* value: NS, non-significant, *P<0.05).

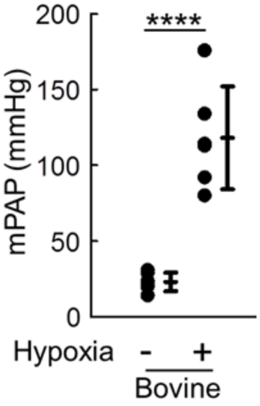

Supplementary Figure 27. PH phenotype of newborn calves exposed to 2 weeks of hypoxia. Higher mean pulmonary arterial pressure (mPAP) in 1 day-old male Holstein calves exposed to either normoxia control (Denver altitude; P_B = 640mmHg) or hypobaric hypoxia (P_B = 445mmHg) for 2 weeks. (n=6 animals per group; mean ± SD plotted; t-test, *****P*<0.001).

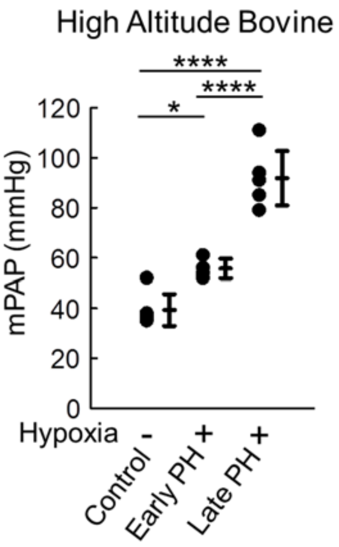

Supplementary Figure 28. Phenotype of naturally occurring PH in adolescent calves at high altitude. Higher mean pulmonary arterial pressure (mPAP) in calves that develop PH compared to non-PH calves at high altitude. The early PH animals are 6-8 months old calves and the late PH animals are 12-13 months old. All animals were born and raised at the same elevation (2200 meters; n=6, 4, and 6 animals / group, respectively; mean ± SD plotted; *P* ANOVA<0.001; t-test, **P*<0.05, *****P*<0.001).

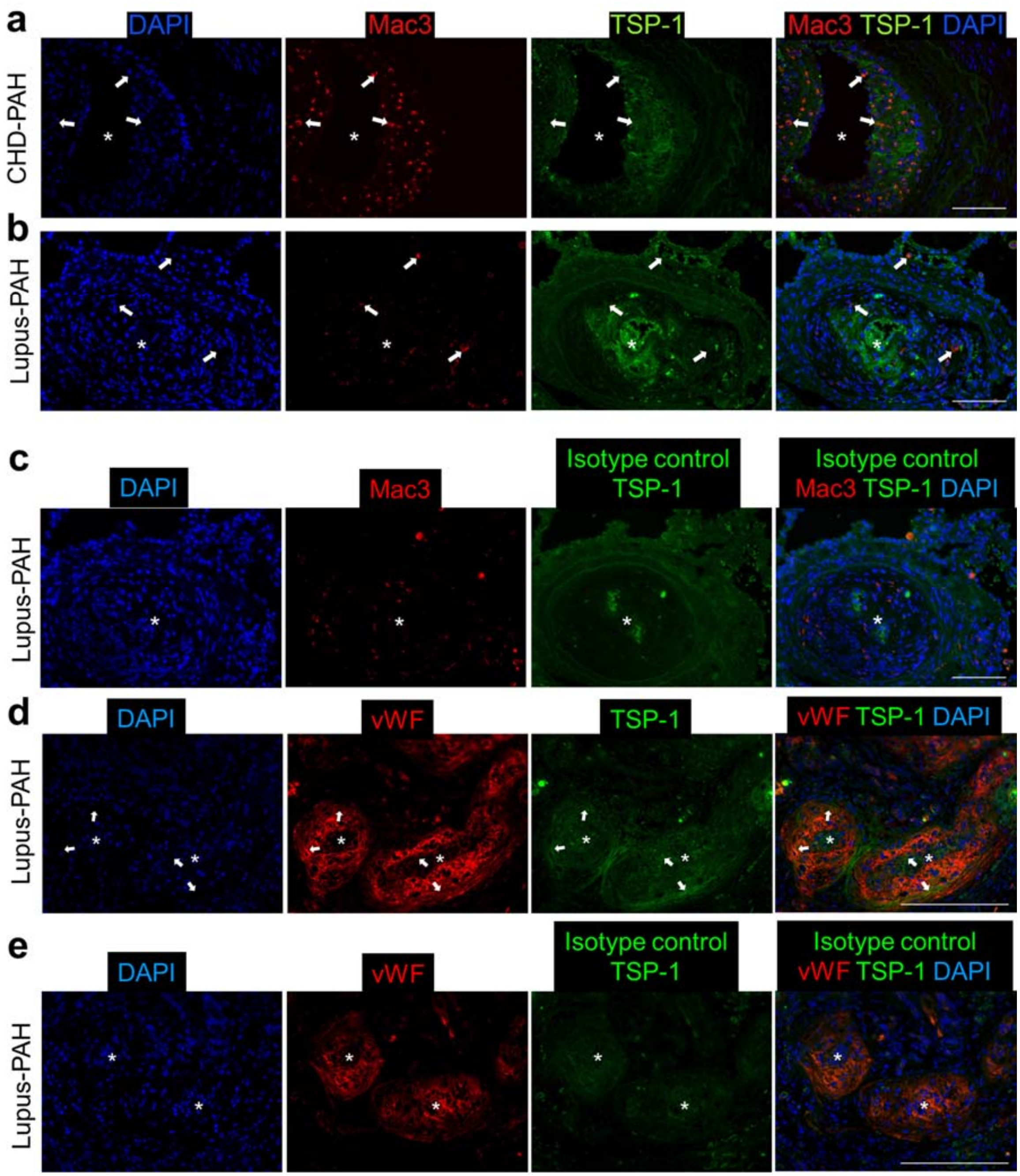

Supplementary Figure 29. Immunostaining for TSP-1 in human explanted lung tissue identifies positive vascular signal in a case of lupus-associated PAH and a case of congenital heart disease-associated PAH. The following samples were immunostained: PAH: 3 scleroderma-associated PAH, 2 lupus-associated PAH, and 1 rheumatoid arthritis-associated PAH; and Control: 2 failed lung donors, both with isolated

cerebral injury. Little TSP-1 immunostaining was observed in the 5 control samples, 3 scleroderma-associated PAH samples, 1 of the lupus-associated PAH samples, and 1 rheumatoid arthritis-associated PAH sample. (**a**) Co-staining for CD68 (macrophage marker) and TSP-1 in a congenital heart disease-associated PAH case (arrows show areas of TSP-1$^+$ staining). (**b**) Co-staining for CD68 and TSP-1 in a lupus-associated PAH case (arrows show areas of TSP-1$^+$ staining). (**c**) Co-staining for CD68 and an isotype-control for TSP-1 (rabbit IgG) in the same lupus-associated PAH case, from an adjacent section to the image shown in (b). (**d**) Co-staining for von Willebrand factor (vWF) and TSP-1 in lupus-associated PAH (arrows show areas of TSP-1$^+$ staining). (**e**) Co-staining for vWF and an isotype-control for TSP-1 (rabbit IgG) in lupus-associated PAH, from an adjacent section to the image shown in (d). (*: vessel lumen; all scale bars 100μm.)

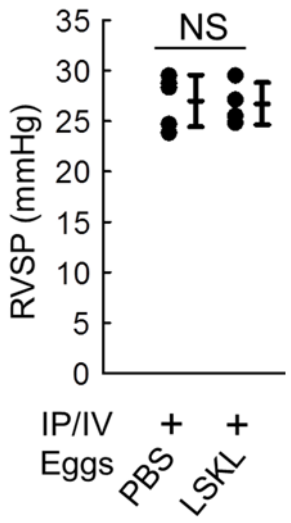

Supplementary Figure 30. No difference in RVSP with a lose dose of LSKL (3mg/kg) treatment in *Schistosoma*-exposed wildtype mice. (n=5 and 4 animals / group, respectively; mean ± SD plotted; t test, *P*=NS, non significant). IP/IV=intraperitoneal and intravenous *S. mansoni* eggs.
